# Special Issue “Ecology of Influenza A Viruses”: Editorial

**DOI:** 10.3390/microorganisms11051287

**Published:** 2023-05-15

**Authors:** Maria Alessandra De Marco, Mauro Delogu, Claudia Cotti

**Affiliations:** 1 Italian Institute for Environmental Protection and Research (ISPRA), 40064 Ozzano dell’Emilia, Italy; 2 Wildlife and Exotic Animal Service, Department of Veterinary Medical Sciences, University of Bologna, 40064 Ozzano dell’Emilia, Italy

Wild aquatic birds constitute the main natural reservoir of the influenza A virus (IAV) gene pool, from which novel IAVs can emerge to infect other animals including avian and mammalian species. These spillover events can lead to variable degrees of viral adaptation in new hosts, ranging from sporadic cases to the sustained circulation of species-adapted IAV lineages, such as those in poultry, humans, pigs, horses, and dogs. In particular, the plasticity of the IAV genome, which is encoded by eight RNA segments, underlies its ability to rapidly acquire—through genetic drift, reassortment, and recombination—mutations enabling adaptation to hosts. So far, sixteen hemagglutinins (from H1 to H16) and nine neuraminidases (from N1 to N9) have been recognized in avian influenza viruses (AIVs) circulating in waterbirds, harboring in their populations the subtype diversity provided by the reassortment of genes encoding these surface glycoproteins [1,2,3,4].

From an ecological perspective, AIVs are natural components of wetland ecosystems, in which they occupy trophic niches represented by susceptible hosts while interacting with other biotic and environmental components. Thus, these RNA viruses have developed adaptive evolutionary strategies to survive and perpetuate in reservoir cells in a context where the ecology of environmental transmission (e.g., subtype-specific durability in aquatic habitats) plays a crucial role in the evolutionary biology of AIVs [5].

For many years, it has been known that the IAV gene pool circulates in avian reservoir populations as low-pathogenic avian influenza viruses (LPAIVs), which, with respect to the H5 and H7 antigenic subtypes, can occasionally mutate in poultry into highly pathogenic (HP) AIVs, which are feared for their potential impact on animal and public health [6]. However, recent years have witnessed the increasing involvement of wild birds in HP avian influenza (AI) caused by the A(H5N1) viruses of the clade 2.3.4.4b belonging to the A/goose/Guangdong/1/1996 (Gs/GD/96) HP H5 lineage, which arose in 2020 from previously circulating A(H5Nx) viruses and spread principally via migratory birds to many regions in Africa, Asia, and Europe [7,8], showing the ability in late 2021 to cross to North America and subsequently South America in the autumn of 2022 [8,9,10]. These ongoing HPAI epidemics have led to unprecedented numbers of deaths in wild birds and devastating outbreaks in commercial and backyard poultry. Moreover, the increasing spillover events from birds to wild carnivore species and the mutations recognized in the virus polymerase protein PB2 during HP A(H5N1) virus infections in Red Fox (*Vulpes vulpes*), Western Polecat (*Mustela putorius*), Eurasian Otter (*Lutra lutra*), and American Mink (*Neovison vison*) pose potential public health issues [7,11,12].

This Special Issue on the “Ecology of Influenza A Viruses” aims to contribute to the knowledge of the transmission dynamics of IAVs and contains six research papers presenting direct and indirect evidence of infection in avian and mammalian species (including humans). In the first research paper, Gulyaeva et al. [13] reported the characterization of two LP H7N3 AIVs isolated from two mallards (*Anas platyrhynchos*) while processing 559 cloacal swabs collected from Anseriformes and other bird species. Sampling was carried out during the annual autumn migrations in 2017 and 2018 in the Caspian Sea region, where major migration flyways overlap, thus connecting Asian, European, and African regions. These two novel viruses contained an H7 hemagglutinin gene phylogenetically related to contemporary strains from Mongolia, Georgia, and Ukraine and predated the detection of this H7 LPAIV sublineage in East Asia in 2019, but more importantly, their polymerase and nucleoprotein segments clustered with contemporary H5 HPAI (clade 2.3.4.4b) viruses, indicating the H7 LPAIV subtype’s large dispersal and its potential for gene reassortment. A longitudinal analysis of influenza A(H5) sero-surveillance performed in ducks reared outdoors in Myanmar is the second research article published in this Special Issue, which was written by Mon et al. [14]. This sero-surveillance study was carried out between 2006 and 2019 on unvaccinated domestic ducks by collecting 78,804 samples from 1817 farms/flocks in selected States and Regions of Myanmar with the aim of confirming H5 AIVs’ circulation and assessing the temporal and spatial distribution of infections. Hemagglutination-inhibition (HI)-positive test results—likely indicating infection with Gs/GD/96-lineage H5Nx HPAI viruses—were obtained every year with annual proportions ranging from 7.1% to 77.2%, thus suggesting the persistent, but intermittent, circulation of Gs/GD/96-lineage H5Nx HPAI viruses in domestic ducks and possible bi-directional spillover between domestic and wild birds. Next, the article published by De Marco et al. [15] assessed serological evidence of AIV infections in 57 bird-exposed workers operating between 2005 and 2006 in an Italian geographic area characterized by both an abundance of wetlands, providing a habitat for AIV reservoir species, and the presence of small-scale poultry farms, representing a potential wildfowl–poultry interface. According to a One Health approach, AIV surveillance data from 3587 birds concurrently reared in this area were also integrated and analyzed in this study. Overall, using an HI test confirmed via a virus microneutralization assay, specific antibodies to AIVs of H3, H6, H8, and H9 subtypes were found in three poultry workers, and double-seropositivity for anti-H11 and anti-H13 antibodies—which were still measurable in October 2017—was detected in one wildlife professional, emphasizing the occupational risk posed by zoonotic AIV strains. The fourth contribution, written by Gobbo et al. [16], reported the results of the targeted active surveillance of H5 HPAIVs in 823 hunted and 521 trapped wild ducks—the latter including 60 Eurasian teals (*Anas crecca*) that were recaptured several times—sampled during the 2020–2021 wintering period. At weekly intervals, the authors collected oropharyngeal, cloacal, and feather swabs (OS, CS, and FS) from each sampled duck for testing using molecular methods for IAV detection, characterization, and pathotyping. Several H5N8, H5N5, and H5N1 HPAI viruses were detected, with the higher prevalences of infection with HPAI H5 clade 2.3.4.4b viruses observed in captured Eurasian teals from November to December 2020 (up to 27.1%), while hunted dabbling ducks, mainly Eurasian wigeons (*Mareca penelope*), showed peaks of infection prevalence in November 2020 (8.9%) and January 2021 (10.2%). Strikingly, all the HPAI-positive ducks appeared clinically healthy when recaptured weeks later, showing the importance of recapture data for following the progression of an HPAI H5 infection in naturally infected wild waterfowl. Moreover, the highest detection efficiency of HPAIVs obtained from the OS and FS emphasizes the benefit of collecting samples from body surfaces, as this process is considered an environmental indicator of the presence of AIVs in water. The fifth study, which was conducted by Christie et al. [17], analyzed White Ibis (*Eudocimus albus*), a nomadic wading bird species, which, through its increased use of anthropic habitats, has recently established several urban breeding colonies in South Florida, the United States. Regarding AI, white ibises have previously been shown to be susceptible hosts for multiple AIV subtypes either through the examination of experimentally infected individuals or as assessed through seroconversion documented in adults in natural populations. To determine whether nestling ibises were infected with AIV and whether AIV dynamics in this age group were influenced by the Florida landscape, 115 ibis nestlings were captured on a weekly basis for 1–4 weeks from urban and natural settings in 2020 and 2021. All choanal/cloacal swabs collected tested AIV negative via virus isolation, whereas maternal antibodies to AIV nucleoprotein were detected via ELISA (with varying rates of catabolism) in 95% of the sampled nestlings. These results confirm that the prevalence of maternal antibodies was reflective of higher adult seroprevalence than previously documented and that nestlings in breeding colonies may have some degree of protection against AIV infection. In the last article of this Special Issue, De Marco and collaborators [18] report long-term serological investigations of IAV in a free-ranging wild boar population under selective control in a protected area of Northern Italy. It is well known that pigs play a crucial role in IAV ecology [19]. From an ecological viewpoint, pigs (*Sus scrofa domesticus*) and their ancestor the Wild Boar (*Sus scrofa*) coexist and share interbreeding potential and susceptibility to pathogens, including IAV. In this study carried out between 2007 and 2014, an overall IAV seroprevalence of 5.5% was detected via ELISA (145 seropositive/2618 tested wild boars), with 56.7% of the screened sera (80/141) testing positive according to an HI assay for the presence of antibodies against the four major swine IAV (sIAV) lineages circulating in Eurasian and Italian swine (sw): avian-like swH1N1, pandemic-like swH1N1, human-like swH1N2, and human-like swH3N2. In particular, antibodies to the prevalent H1N1 subtypes were detected from 2009 to 2013, and H3N2 subtype seropositivity was found in six years (2007, 2009–2012, and 2014), whereas H1N2 subtype antibodies were only detected in 2012. The obtained findings, including H1N1 and H3N2 seroconversions from three of twenty-seven wild boars recaptured one or more times, suggested the occurrence of sIAV spillover events from pigs to wild boars sampled in an anthropized study area.

In the context of the rapid climatic and environmental changes occurring in the Antropocene, the increasing involvement of wild birds in H5 HPAI (clade 2.3.4.4b) viruses circulation continues to pose a threat to animal and public health worldwide [7]. Consequently, the complexity of ecosystem connections requires a holistic approach, allowing for the higher clarification of the biotic and abiotic driving forces that influence global IAV circulation [20,21]. We are confident that the ecological approach characterizing this Special Issue’s contributions will provide useful insights into the implementation of the ever-growing IAV surveillance programs.
